# Beyond survival mode: Organizational resilience capabilities in nonprofit arts and culture fundraising during the Covid‐19 pandemic

**DOI:** 10.1002/nml.21524

**Published:** 2022-06-10

**Authors:** Marta Herrero, Simone Kraemer

**Affiliations:** ^1^ Department of Theatre, Film, Television and Interactive Media University of York York UK; ^2^ Development Office University of Kent Kent UK

**Keywords:** arts and culture organizations, Covid‐19, nonprofit fundraising, resilience capabilities

## Abstract

Organizational resilience remains an under‐explored topic in the nonprofit management literature (Searing et al., 2021). Despite an increasing number of studies framed by management perspectives and organizational theory, their focus is on how forprofit organisations react against external crisis by developing ‘resilience capabilities’; ways of understanding and of working designed to reduce uncertainty and restore balance to the organisation (Lengnick‐Hall, et al., 2011; Williams et al., 2017). This article draws on literature on ‘resilience capabilities’ and on in‐depth interviews with nonprofit fundraisers, carried out during the early stages of the Covid‐19 pandemic, to examine how they compensated for a sudden drop in financial revenue by devising alternative, strategic ways of fundraising. It argues that non‐profit fundraisers deployed cross‐capability building, which combined and merged emotion‐related and behavioural capabilities to achieve strategic, practical results.

## 1 INTRODUCTION

Organizational resilience remains an under‐explored topic in the nonprofit management literature (Searing et al., 2021). Despite an increasing number of studies framed by management perspectives and organizational theory, their focus is on how forprofit organizations react against external crisis by developing “resilience capabilities”; ways of understanding and of working designed to reduce uncertainty and restore balance to the organization (Lengnick‐Hall et al., 2011; Williams et al., 2017). This article draws on literature on “resilience capabilities” to examine the extent to which it can help explain how fundraisers in the nonprofit sector operate and work during an external crisis such as the Covid‐19 pandemic. The analysis is based, predominantly, on qualitative data, in‐depth interviews carried out with fundraisers between September and November 2020; a time when public health restrictions were introduced in the UK, and its population were to follow a stay‐at‐home government policy to mitigate the spread of coronavirus. The article investigates how fundraisers in nonprofit organizations devised alternative, strategic ways of generating income to their usual fundraising practices, to compensate for a sudden drop in financial revenue. It argues that fundraisers deployed cross‐capabilities which combine and merge existing capabilities in order to achieve strategic, practical results.

The financial situation experienced by arts and culture nonprofits in this article is a direct result of the UK Government's austerity policy which goes back to 2010. This was set out to reduce public spending to the sector, translated into a drastic reduction in direct funding to arts and culture nonprofit organizations/activities (Harvey, 2016), and reduced local councils' ability to support these. In 2010, local councils' expenditure on arts and culture decreased by £400 million, and, by 2015, spending on the arts and culture sector in England declined by 16.6% (Harvey, 2016). In 2019, another wave of financial cuts to government spending on local authorities was announced, which saw their income further reduced thus affecting the activities of local museums, libraries, and art festivals.^1^ It is thus not surprising that the nonprofit arts and culture sector has been deemed as one of the worst affected by the Covid‐19 pandemic, and one of the least likely to recover (Radermecker, 2020). In March 2020, the introduction of public health, national restrictions to mitigate the spread of coronavirus prohibited all public‐facing activities, including revenue generating events, from ticket sales at live performances and exhibitions, to any face‐to‐face fundraising (corporate entertainment, gala dinners, and fundraiser events). This was a period of great instability and change, characterized by enforced social distancing, gradual easing of restrictions, including the reopening of indoor theaters, followed by a second national lockdown at the end of October 2020.

The article offers a novel theoretical contribution to nonprofit resilience research focused on the work of fundraisers, applying insights from the literature on resilience in nonprofit and forprofit environments framed within human resource management, organizational studies, and crisis management. It compiles a typology of resilience‐building capabilities in both pre‐crisis and crisis environments (Boin et al., 2010; Bundy et al., 2017; Williams et al., 2017) which is tested and applied to the work of nonprofit fundraisers. Its underlying premise is that in the process of resilience‐building capabilities, reformulated types of knowledge and understanding come to the fore, which help fundraisers learn about alternative ways of doing their work in challenging environments ridden by uncertainty and a lack of routine. We thus treat fundraising as an evolving form of knowledge that is embedded, emerging and inherent to practice/s (Billett, 2001; Gherardi, 2000, 2009; Ibert, 2007; Nicolini et al., 2003; Orlikowski, 2002; Schatzki et al., 2001). This perspective differs from an understanding of fundraising as a set of rules and a priori guidelines to be learnt (Sargeant & Jay, 2014), or as a disposition of actors (Bourdieu, 1984). The article contributes to the literature on nonprofit organizational resilience by arguing that resilience capabilities, when examined against the work practices of nonprofit fundraisers in the arts and culture sectors, display distinct differences. Our findings indicate that fundraisers combined and merged some of the actions and behavior present in the resilience capabilities literature. We thus propose the term “cross‐capabilities” to capture the strategic ways in which fundraisers deployed resilience‐related practices, which they adapted to address and surmount their specific work circumstances.

The article is structured as follows. Firstly, it introduces a contextual overview of the main issues affecting nonprofit arts and culture fundraising in the UK. Secondly, it builds an analytical framework on fundraising research with insights from resilience studies in organizational environments ridden by crisis and uncertainty. Thirdly, the methodology section describes the process of qualitative data collection and analysis. The following, fourth section examines the qualitative data on the work practices of fundraisers during the Covid‐19 pandemic vis‐à‐vis the typology of resilience capabilities drawn from the literature. The article concludes by arguing that fundraisers' resilience‐building strategies follow a pattern of cross‐capability building characterized by the combination of capabilities, and it elaborates on the implications of the research for future fundraising practice.

## 2 A BUILDING‐RESILIENCE CAPABILITIES FRAMEWORK FOR ARTS AND CULTURE NONPROFIT FUNDRAISING DURING COVID‐19

Scholarly fundraising research follows two main paradigms. On the one hand, fundraising is treated as a “distinct knowledge base acquired through sustained periods of training and education”; acquisition of such knowledge is of primary importance to “professionalize” one's practice (Breeze, 2017, p. 168). Usually written from a marketing or management orientation, this literature is designed to help fundraisers “get the job done” (Holman & Sargent, 2006; Lloyd, 2006; Sargeant & Jay, 2014). On the other hand, an emerging field of critical fundraising studies (Alborough, 2017; Breeze, 2017; Herrero & Kraemer, 2020) is gradually building a theoretical base to examine the “taken for granted”, “common sense” assumptions underpinning fundraising, as something that one learns “by just getting on with it” (Breeze, 2017, p. 94). In this article, we argue that the organizational environment within which fundraisers operate is crucial to their in/ability to carry out their work effectively. Rather than treating organizations as environments where fundraisers simply put into practice their repertoire of pre‐existing knowledge, we envision that knowledge about how to fundraise occurs through and emerges out of organizing; an ongoing process of practice and learning where existing ways of thinking and working are re‐thought and re‐configured to adapt to internal and external situations and environments.

Our approach to fundraising research is framed within the practice‐based studies (PBS) perspective (Herrero & Kraemer, 2020). Since its emergence in the 1990s, it has increasingly become one of the established ways of analyzing how learning occurs within organizational environments (Billett, 2001; Gherardi, 2000, 2009; Ibert, 2007; Nicolini et al., 2003; Orlikowski, 2002; Schatzki et al., 2001). Shifting the centre of analysis from individuals and their actions towards an understanding of knowledge as inherent in practice, PBS takes a processual view of organizations (also referred to as organizing), which are seen as made up of **“**connections between and among actions and of the knowledge/s they help constitute.**”** The study of such connections and actions illustrates how knowing is sustained in and manifests itself through practice. Thus, knowledge occurs in and through action, through organizing. It is always situated, negotiated, and embedded, rather than a stable disposition of actors. Only when practices become “stabilized,” organized around shared practical understandings, can they facilitate the construction of actors' identities (Czarniawska, 2013, p. 14).

A fundraising as practice perspective helps ground our analysis of how organizations develop “resilience capabilities” (Lengnick‐Hall & Beck, 2005; Williams et al., 2017) in the work fundraisers do. The term “resilience” is commonly used to designate individual and organizational practices responding positively to a setback or crisis in various contexts (Hickman, 2018). Despite critiques of resilience as an ideological “smokescreen” that shifts “the onus into individuals and communities to take care of their own problems” (Dayson et al., 2021, p. 297; see also Bene et al., 2012; MacKinnon & Derickson, 2013; Newsinger & Serafini, 2021), research on resilience continues to rapidly increase on a par with the substantial disruptions – from terrorist attacks to financial downturns ‐ that create an environment of uncertainty and crisis (Lengnick‐Hall & Beck, 2005). When an organization demonstrates a collective capability for resilience, it means that it has an ability to understand “the current situation”, as well as “to develop customized responses” that reflect such “understanding” (Lengnick‐Hall & Beck, 2005, p. 750). The ability to understand and to respond are carried out at the level of practice, in the interactions of individuals within the organization and with external actors. Such practices are continually evolving and ongoing within environments affected by differing degrees of uncertainty and crisis. In what follows, we draw on literature on resilience responses to outline the various resilience capabilities that are carried out at the level of practice, and whose presence, or absence, can help assess the extent to which organizations, collectively, are able to exercise resilience

***Cognitive capabilities*** indicate an understanding, or “constructive conceptual orientation”; a combination and deployment of knowledge and repertoires of action, for example, vision, sense of purpose, strong values and deep knowledge and expertise, to be applied to the resolution of problems (Lengnick‐Hall & Beck, 2005). They enable people, groups, and organizations to “manage what they know,” noticing and making sense of signals of potential disruptions (Lengnick‐Hall et al., 2011). An aspect of cognitive capabilities is the existence of a “strong ideological identity” that is core and value‐ridden and offers a “prime directive for organizational choices” (Lengnick‐Hall & Beck, 2005, p. 751). ***Behavioral capabilities*** designate both practical action alternatives as well as a repertoire of “complex and varied action inventories” that are able to steer a “dramatically different course of action from that which is the norm” (ibid: 750–01). Behavioral and cognitive capabilities are intricately linked. For example, organizations with “myopic understandings” of work tasks are more likely to experience recurrent problems, and thus be less resilient. Small firms are also more likely to implement creative action to help maintain positive functioning than large firms (Williams et al., 2017, p. 744). Overall, flexible, and wide‐ranging thinking coupled with access to structures that facilitate the rapid implementation of changes encapsulate an ideal behavioral response to pre‐adversity and adversity situations. ***Relational capabilities*** (Lengnick‐Hall & Beck, 2005) refer to access to and exchange of resources that can enhance an organization's positive functioning in the face of adversity. A case in point are social networks amongst and between organizations that can facilitate access to resources such as information, loans, and gifts. Being part of a network, and the existence of trust within relationships, can enable resilience whilst a lack of access to networks and relationships can have the opposite effect (ibid, p.752). ***Emotion‐regulation capabilities*** are the mental fortitude capabilities that provide actors with “mental hardiness and self‐regulation enabling them to cope with adverse situations” (Williams et al., 2017). The presence of individual and/or collective optimism, hope, as well as having opportunities to express and discuss emotions, is likely to enhance emotion‐regulation capabilities. In turn, emotion‐regulation capabilities are likely to increase the accuracy of judgments and assist in the anticipation of and preparation for the unexpected.

Having outlined the main resilience capabilities and how these are carried out at the level of practice, in what follows, we explain the methodological decisions and process underpinning our collection of data and analysis.

## 3 ARTS AND CULTURE NONPROFIT FUNDRAISING: RESEARCH METHODOLOGY

The research presented here was designed to gain in‐depth understanding of how fundraisers adapted their existing ways of working, and the tools, (or lack of them), they had at their disposal to deal with the challenges of fundraising during a pandemic. The analysis is based on qualitative data collected from semi‐structured, in‐depth interviews carried out between September and November 2020. Interviewees were recruited via a survey ‐ a collaboration between the Chartered Institute of Fundraising's (CIoF) Culture Sector Network, the University of Sheffield, and the University of Kent ‐ that took place in early August 2020 (Herrero et al., 2021). This was a period of gradual easing of social restrictions, including the reopening of indoor theaters, and the government backed “Eat Out to Help Out” scheme offering discounts on meals.^2^ The survey was widely distributed by the CIoF via social media, and by email to CIoF's newsletter groups, professional networks, volunteers, and Culture Sector Network's members.

The data collection followed a convenience sampling approach. It is estimated that the survey reached just over 1000 potential respondents. A total of 106 responses were collected ‐ a response rate rather low, of just over 10% and 91 were deemed valid. The CIoF's involvement in the distribution of the survey meant that participants were recruited from within its close networks. A weakness of this closeness is a potential bias towards generating responses from members of the CIoF's network, and thus a lack of representation of arts and culture fundraisers in the UK, more widely. Equally important is the fact that high response survey rates are desirable and seen as an indicator of data quality and representativeness. Given that the findings emerging from this data only apply to the qualitative sample derived from a survey with a rather low response rate, we cannot generalize about the working practices of arts and culture fundraisers in the UK. However, surveys achieving low response rates, such as this one, are nonetheless representative of certain attitudes, for example, within the fundraising workforce, that cannot be dismissed as uninformative (Meterko et al., 2015). This type of sampling also gave researchers the advantage of being able to collect, within a relatively short period of time, and with very limited resources, quantitative and qualitative responses from fundraisers whose work had been affected by the pandemic.

One question in the survey asked respondents if they would agree to take part in further research by agreeing to be interviewed about their work practices during the pandemic. 20 survey respondents agreed to be interviewed, and 18 participated in the final interview process. The final sample included 15 full‐time fundraisers, 2 consultants, and 1 volunteer. 9 interviewees were fundraising directors, involved in formulating fundraising strategies, liaising with funding partners and overseeing all fundraising activities; and 9 worked in a fundraising management capacity, organizing fundraising events, developing donors, managing budgets, and working towards fundraising targets. All interviews were conducted online, via Zoom, and lasted, on average, 1 hour; audio recordings were saved for transcription purposes. Interviewees worked mostly in the performance arts sector (music, theater, and ballet), in art museums, and in heritage organizations. In terms of the total fundraised income per organization, of the 18 interviews, 2 were consultants and did not respond to the question about their organization's fundraised income. Of the 16 remaining, 4 worked in organizations with an income between £1 M‐£4.99; 4 in organizations whose income ranged between £500,000 and £999,000; 7 fundraisers worked in organizations with a fundraised income between £100,000 and £499,999 and 1 fundraiser for an organization with an income between £10,000 and £99,000.^3^ 8 interviewees had been fundraising from 0 to 3 years, 8 from 4 to 10 years of fundraising work experience, whilst only 2 interviewees had been working in fundraising for more than 10 years.

We followed Denscombe's (2007) five steps methodology and a thematic approach to our data analysis (Braun & Clarke, 2006), designed to enhance the credibility and thus trustworthiness of our data, especially when it referred to “investigator triangulation”; the two authors were equally involved in the coding, analysis, and interpretation of the findings. We started with 1‐“data preparation” – generating textual transcripts ready for analysis; 2‐“familiarity with the data” ‐ involved initial readings of the transcripts undertook by each of the two authors separately to identify thematic areas of intervention and action by fundraisers; 3‐“interpreting the data” ‐ we undertook analysis of identified themes (Braun & Clarke, 2006) to which we assigned specific resilience‐building capabilities following our theoretical framework; 4‐“verifying the data” ‐ this stage involved comparison of data findings between the two authors, and further analysis to expand on initial set of resilience capabilities; and 5‐“showcasing the data” ‐ we produced a narrative detailing the opportunities and challenges experienced by fundraisers when building resilience capabilities

Initially, we sought to explore whether any of the working practices of fundraisers confirmed the existence of any of the resilience capabilities outlined in the literature review, and which are deployed in times of adversity and crisis within organizational contexts. Our initial analysis showed instances of resilience, which confirmed the categories developed in the literature review. But it also indicated the deployment of cross‐capabilities where fundraisers worked with and across two types of resilience to build a combined form of capability. We thus decided to conduct further analysis of cross‐capabilities to help develop in more detail how resilience is built up and negotiated in and through a combination of practices. In this second stage, we sought to establish the extent to which instances of cross‐capabilities were applicable to all types of resilience, or only to a selected few. Thus, further analysis was focused on finding instances of cross‐capability examples of resilience, as well as instances of single capabilities

## 4 ANALYSIS – IMPLEMENTING RESILIENCE CAPABILITIES IN ARTS AND CULTURE FUNDRAISING DURING THE COVID‐19 PANDEMIC

In this section, we present the findings from our analysis framed by the list of resilience‐building capabilities identified earlier on. We start by identifying the three main capabilities fundraisers work on building during the pandemic, cognitive, behavioral, and relational. However, when it came to identifying emotion‐related capabilities, our evidence suggests that this a cross‐building category; a practice deployed to support and strengthen the building of behavioral capabilities. Thus, in the last part of this analysis, we refer to emotion‐related capabilities as an instance of “cross‐capability building.”

As a way of contextualizing the work of fundraisers, it is fitting to refer to the ways the organizations referred to below fundraised and generated commercial income. Aside from differences in organizational size and amount of fundraised income, there are some clear general patterns in the ways nonprofits raise income. Fundraised income comes primarily from membership programs to which individuals and businesses can subscribe, usually on an annual basis. In general, these programs offer, in return, participation and attendance to members‐only events and activities, and discounted tickets. Fundraising also involves asking for donations, and, in these cases, individuals and businesses who choose to donate do so outside the framework of any membership programs. Another type of fundraised income is business sponsorship, which was drastically reduced during the pandemic. As some fundraisers below indicate, businesses' ability to operate as usual was hampered by the pandemic, and at such uncertain times their participation and contribution to nonprofits was quickly withdrawn. Overall, the main way in which fundraising was affected, and which applies to all the types of raised income – sponsorships, memberships, and donations – lies in the fundraisers' inability to conduct face‐to‐face activities and meetings with their (prospective) sponsors, members, and donors. Without such close contacts and interactions, fundraising had to be re‐imagined, and this meant that fundraisers had to create new ways of connecting, and interacting with their supporters.

### 4.1 Cognitive capabilities

As mentioned earlier, cognitive capabilities refer to the existence of an understanding, of knowledge and repertories of action, such as vision and sense of purpose, that are applied to the resolution of problems (Lengnick‐Hall & Beck, 2005). In explaining their thinking about the effects of the pandemic on fundraising, interviewees shared a clear understanding of what changes needed to be implemented to fundraise most effectively. Our survey data shows that 30% of fundraisers were finding out more about the sorts of experiences donors would like from them, and 24% said they were spending time find out more about why donors support their organization. This engagement is also evident from our interviews which demonstrated a degree of flexibility from fundraisers as they re‐thought how to communicate and interact with donors. However, when asked about the expected impact fundraisers thought the pandemic would have on their fundraising, 62% said that they expected to bring lower income.

Reassessing the type of messages used to communicate with their stakeholders was deemed a priority. Rethinking their use of social media, fundraisers thought that their organization's charitable role, rather than their function as a provider of cultural offer, should be the focus of any stakeholder engagement. In pre‐pandemic times, social media was used as a marketing tool to promote shows and performances, but now “we were ready to say we are a charity” (FR6). Fundraisers were aware that any desired change in behavior from their donors, e.g., increasing or continuing with their support, and from the public more generally, would have to be preceded by a renewed understanding (prompted and strategically highlighted by fundraisers) of the organization's charitable status, rather than its commercial operations. For some, “encouraging people to support us” meant “really highlighting” their charitable work: “what we're doing to help groups and societies become more resilient to become stronger” (FR8). However, an interviewee noted that being perceived as a charity could be very challenging because certain art forms were not readily associated with their charitable status. Ballet is “fundamentally just one of the least attractive causes … people are amazed that a ballet company would be a charity.” This was a hurdle that other charities, for example, those in the music sector did not “face in quite the same way” and thus found it easier to attract donors or funders as they were more “interested in music … [but] hardly any … in dance” (FR16).

Fundraisers also reassessed their engagement with donors. They were aware that the pandemic could have changed what donors' thought was “valuable for them” and thus “anything that was valuable before is not seen as valuable” now (FR2). The inability of organizations to deliver a cultural offer was at the forefront of fundraisers' renewed thinking about their donor engagement and stewardship. Even though strengthening engagement with existing donors was seen as “a reliable path to follow” (FR4), rather than seeking to engage new donors, their physical remoteness from the organization was deemed problematic. Potentially, donors could change their priorities and decide to give their money to “vulnerable households” or to a “women's refugee”, rather than continue with their ongoing commitment to the arts (FR2).

Ethical fundraising and decision making. We found evidence that fundraisers changed their thinking about asking for money during the pandemic because they felt it was a sensitivity issue. “Doing the right thing at the right time” was more important than asking for money when the time was not deemed to be “right.” A fundraiser explained a change in plans to launch a legacy campaign that had secured sponsorship support from a law firm. With the “death rate being so widely publicised,” it was felt that this was not the “right time” to think about legacies (FR6). Similarly, a fundraiser referred to the decision not to launch any fundraising appeals on the grounds that “it just never felt appropriate” at a time when other “frontline causes are needing attention.” Also deemed “inappropriate” was to ask certain major gift prospects for financial support, especially producers working in the theater sector, who were “facing a lot of financial uncertainties themselves or are trying to save the industry” (FR15). Even though such decisions were mostly agreed upon, they also led to disagreements. One fundraiser who was asked to put all fundraising efforts on hold for four months because the CEO and the board of trustees were “very anxious about fundraising and asking people for money,” voiced her disagreement with the decision; however, after “a huge argument,” she noted, “I lost” (FR9).

A renewed awareness of the changes affecting the external funding environment and thus their relationships with funders was also part of fundraisers' reassessment of their engagement with charitable trusts, foundations and the government as sources of income. An interviewee referred to the reduction in the number of funders available, noting that they “are just completely overwhelmed with people at the moment. So, I don't hold out much hope” (FR3). The impact of many “trusts and foundations” pausing their “normal grant giving” was deemed worse than if individual donors had decided to “drop out,” and stop their donations. A fundraiser described the situation as being “stuck” but with the expectation that, in time, the situation may improve. Her organization could not apply for further funding because they were unable to deliver on their current projects which were already in receipt of financial support: “[we cannot] go back to for anything else because they have already given us the money. We cannot start work; we are hoping to start work in the spring” (FR5).

### 4.2 Behavioral capabilities

The term behavioral capabilities is used to designate practical action alternatives that can steer a “dramatically different course of action from that which is the norm” (Lengnick‐Hall & Beck, 2005, pp. 750–751). An example of fundraisers' deployment of behavior capabilities was found in the course of action they took around the issue of engagement with (new) supporters. Our survey showed that 54% of fundraisers were prioritizing engaging with existing supporters, and 38% responded that their focus was on both a mixture of existing and new supporters. Whilst there are different strategies, they nonetheless demonstrate that fundraisers found it fundamental to take practical action about their donor engagement strategies. These findings were supported by our interview data where most fundraisers indicated their priority towards a donor retention strategy. We thus describe prioritizing donor retention, rather than attracting new donors, as a behavioral capability and building strategy. The “uncertainty” caused by the pandemic coupled with a lack of resources led to a reliance “on the kind of goodwill and commitment” from existing donors (FR4). Fundraisers prioritized “the reporting and the stewardship of our existing supporters as good, as best as we can” (FR15), rather than seeking new sources of income (FR7). A fundraiser explained how her team designed a campaign to keep supporters interested in the organization. Each staff member was allocated between 10–20 supporters whom they would phone and have a chat with, resulting in 150 phone calls approximately. She emphasized the importance of such stewardship, which “wasn't about money”, but about “keeping [supporters] interested in the organization” (FR4). However, our data also showed that not all organizations were able to maintain such a level of individualized support. A fundraiser explained that when she was put on furlough all relationship building with donors stopped, as well as any conversations with donors who were ready to “help in any way” (FR6). Another fundraiser explained that her organization prioritized “back‐to‐back bid writing”, which meant that she was unable to spend time maintaining existing relationships with donors (FR2).

A related example of behavioral capabilities was the use of digital technology in donor retention strategies; a finding also reflected in our survey data with 65% fundraisers saying that they provided donors with access to online activities. During the interviews, a fundraiser explained how the decision to deliver online training to local youths was part of their donor engagement strategy because it showcased how the organization prioritized its charitable values and mission to existing donors (FR8). From a practical perspective, the use of digital technology helped fundraisers connect with donors that “would ordinarily have had a bit of trouble scheduling in a meeting because they are so busy, but they will squeeze in an online call” (FR5). A fundraiser working for a heritage organization reported an increase in the use of online communication, distributing their e‐newsletters to members weekly, rather than monthly, as a way of sharing “lots of lovely photos of wildlife.” For some members this “was their way of accessing the outside world” (FR14). However, a fundraiser explained the need to be age‐sensitive when using digital technology to interact with donors. For those over the age of 50 Zoom meetings could be “very stressful.” But when she spoke with donors over the phone, and especially when making an ask, it “worked really well” as “everybody seems to relax a little bit more on a phone call”, when they were “not being distracted by a picture of themselves” (FR9). But the use of digital technology had its own challenges. One fundraiser spoke about the digital disenfranchisement of her organization's “main beneficiaries” because they support and work with “10% of the most deprived people in the country” who might not necessarily have online access (FR9).

The delivery of online content and use of social media also helped solve long‐term organizational silos and improve organizational efficiency. A fundraiser described how his organization broke “new ground” by balancing the priorities of marketing and fundraising departments so that ticket sales and fundraising were not seen as separate strategies. In this case, fundraisers and marketing officers worked together to carefully select the most appropriate online channels and platforms, creating Facebook posts and live stream videos to deliver both cultural content and include fundraising asks (FR16).

However, the use of digital technology was inevitably the last and only “viable option”, such as when the furlough scheme reduced the size of a fundraising team from six to two. In this case, organizing digital events with the “artistic director” was the only way to make sure “we remained important in our supporters” lives' (FR15). These views resonate with our survey findings where fundraisers reported an increase in social media use from 12% before the pandemic, to 25% during the pandemic. But the adoption of digital activities was not always successful, especially when not combined with the necessary fundraising skills. An interviewee explained that even though various education teams delivered online activities for children to do at home with their parents, when the fundraising team tried to work with the same education teams to promote fundraising “only one team did raise some money” because they felt “uncomfortable” about asking for money (FR14).

Fundraisers also built new behavioral capabilities by adopting ways of working that led to developing new skills, as well as by making strategic management and leadership decisions. An interviewee explained that they had developed “really useful skills;” her new way of working was reactive, but also involved a “flexibility and adaptability” to the new environment, and “going with the flow.” Working reactively differed from a more patterned and organized way of working characteristic of fundraising prior to the pandemic, which was driven by tight timelines and yearly targets. For the fundraiser, the change was positive, and a way of breaking free from “some of those things we kind of overly hold onto” (FR15).

Fast‐paced innovation was also an outcome of the pandemic. Fundraisers no longer had time to try and test “one or two new fundraising products in a year,” instead, everything “was new and very fast paced” (FR5). Existing skills such as “resilience and perseverance” were “incredibly important” in dealing with the financial uncertainty. An interviewee described “resilience” as “being able to be knocked down and stand up because you didn't get a grant” (FR9). This opinion was shared by another interviewee who argued that fundraisers were well‐suited to deal with the pandemic's uncertainties as they were “more resilient,” used to dealing with rejection, and focused on getting on with their job (FR1).

However, some fundraisers were unable, or experienced difficulties in building skill‐related behavioral capabilities. One challenge was the extra additional work brought about by the pandemic described as “trying to just keep on top of all the coronavirus related things we have to do” (FR14). Another interviewee found his job challenging because it was difficult to know what “everybody else [was] doing [and you were] just kind of winging it and seeing how this year goes” (FR11). For others, the pandemic created a need for new planning and re‐organizing job duties; a fundraising manager explained how she had to spend time with “each team member” and decide what they now needed to do individually, and as a team (FR5).

Overall, however, our survey data indicated a positive outcome in fundraising efforts which we can designate as building behavioral capabilities. 86% of fundraisers who had adopted new approaches and/or amended their existing fundraising activities said that these had met or exceeded their expectations, with only 14% saying that new approaches had not met their expectations.

### 4.3 Relational capabilities

Relational capabilities (Lengnick‐Hall & Beck, 2005) refer to access to and exchange of resources that can enhance an organization's positive functioning in the face of adversity. Evidence of relational capability building was found in fundraisers' ability to successfully draw on their external environment to build up relationships and networks. Having good relationships with funders and artists to support their fundraising were a case in point. It meant that those organizations with existing restricted funding were able to request for it to be made unrestricted, so that it could be used to pay for core activities (FR15). Similarly, having good relationships with artists helped an organization with its fundraising. In this case, a playwright who had gained experience at that organization but was by then well‐known in the television and film industries, worked as a fundraiser for over two months. The playwright got in touch with her industry contacts and made “asks” whilst also explaining the importance of supporting the organization, describing it as the “training ground for … writing talent in a world now where everybody is watching more television than ever” (FR15).

And yet, the survey data demonstrates a rather different picture. When asked about an organizations' ability to secure additional funding from the government, charitable trusts, and foundations 51% of fundraisers reported their organizations were not eligible for local government funding, with only 32% having accessed such funds. 37% reported not being eligible for national government funding, even though 41% were successful in securing such funds. Success rates for securing funding were at their highest for lottery funders, including the Arts Council of England (ACE) with 58% of fundraisers able to access funds, and 55% successfully securing funds from charitable trusts and foundations.

But the interviews revealed the importance of participation in networks. Fundraisers gained increased access to new and existing external networks that helped them gain skills and support. A fundraiser explained how an existing network in the performing arts fundraising community became a “lifeline”, with more frequent zoom meetings every two weeks “just to talk about how things were going.” Even though the group knew each other prior to the pandemic, they got to know each other better so that “myself, my peers, and my team are using and relying more on those sorts of networks.” In this case, it allowed the fundraisers to make new connections that became “quite personally useful,” and he was even able to meet in person with them after the lockdown (FR15). A newly formed group for managers in the art sector was started as a result of the pandemic. The group met periodically and a fundraiser explained that it had been a good experience, “everyone speaks quite frankly” about their challenges, and “logistical issues” (FR16). One fundraiser noted that she “had more connection with colleagues during lockdown than I ever had” (FR2). The connections also spanned over other social media networks such as Facebook groups, Twitter, and direct chats with peers. A fundraiser reported that “everyone's in a different situation, but broadly similar, so that's been really nice to talk to other people and I found that really helpful and I've been trying to encourage that people in my team to do stuff like that” (FR14). Fundraisers shared an understanding of the value of co‐operation, of the need to “support each other and are always willing to share ideas”, helping “each other get along”. There is a sense of a shared community, an interviewee noted: “I love being part of a fundraising world. We will help each other get along” (FR13). However, despite the positive experiences and examples of relational capability building found amongst fundraisers, we also identified examples of a strained relationship, especially between the government and the fundraising sector. Some interviewees pointed out that the government was unable to understand the needs of the arts and culture nonprofit sector, and had not taken the necessary steps taken to address its needs. The fact that some freelancers were not eligible for any self‐employed income support (FR15), and the small allocation of government funding, which was “incredibly poor” and “really small” in comparison with sectors like “aviation, and supermarkets” (FR13), were both cited as reasons for a lack of understanding and adequate government support.

### 4.4 Cross‐capability building: Emotion‐related and behavioral capabilities

In what follows, we elaborate on how emotion‐related capabilities facilitated fundraisers' implementation of behavioral capabilities in what we describe as an example of cross‐capability building. Emotion‐related capabilities initially refer to the presence of mental fortitude that helps individuals cope with adverse situations and is expressed in the form of individual and/or collective optimism and or hope. Having opportunities to express and discuss emotions is also likely to enhance emotion‐regulation capabilities (Williams et al., 2017). So far, our analysis has demonstrated that fundraisers implemented behavioral capabilities as seen in their new strategy to work with existing donors and supporters. But in so doing, they also drew upon emotion‐related capabilities, seen here in fundraisers' prior knowledge of donors who had shown an emotional attachment to the organization. This knowledge led fundraisers to prioritize and target such donors in their approaches.

A fundraiser explained that when donors felt strong emotional ties to the organization, they also shared a sense of membership and inclusion as well as responsibility for the organization's financial well‐being. Whether they were able to “give a lot or a little”, donors saw “themselves as being part of” the organization (FR16). Donors' loyalty, in particular, ensured a steady level of donations, especially at a time when the pandemic made it impossible for such donations to be reciprocated with any face‐to‐face benefits (FR15). Similarly, fundraisers drew upon emotion‐related capabilities in their relationships with funders, as seen in the decision to adopt a “really open and honest approach with our funders … more than we normally do.” An example of honesty was the case of a fundraiser who felt that being open with existing funders, and explaining to them her organization's difficult financial situation, and their fundraising plan for “recovering a loss of income” was the best approach. Her strategy was to ask funders to repurpose some restricted income so that the funds could be spent in “core” expenditures. Such a strategy paid off, as all funders agreed to the request in what the fundraiser described as “an early exercise in honesty” (FR14).

For other fundraisers the challenge was balancing the new requirements of the job, whilst also caring for their own well‐being and that of others. Whilst some fundraisers in leadership roles were able to draw upon existing emotion‐related capabilities by expressing emotions to empathize with others, and/or improve their own well‐being, finding a balance between managing the well‐being and emotions of others as well as their own, was found very challenging. The interviews echoed data collected from the survey were 63% of fundraisers said their workload had increased during the pandemic, and levels of job satisfaction decreased from an average of 8 out of 10 pre‐pandemic to 6 out of 10 during the pandemic. During the interviews, a fundraiser manager who explained that she enjoyed her job, described the pandemic as a time for learning: “learning how to be present, empathetic and supportive, but also … to switch off and look after me” (FR15). Intense workloads led to fundraisers finding their physical and mental well‐being affected. Whilst some were able to take time off and gain some perspective by changing their work routine and implementing “good self‐care” (FR8), not all organizations had the resources to support fundraisers in helping themselves. Some offered online support via email in case of concerns over “mental health or well‐being,” but lacked any personalized contact such as phone calls to check “how I was” (FR18). One interviewee felt her mental health was particularly affected by having to shield and needing people around her. However, even though her employer was aware of the issues, her organization lacked the resources to assist her. Adding that she felt “much better these days,” it was finding her “own resources” that ultimately helped alleviate her situation. She felt that attending the CIoF's annual convention was beneficial because it put her in touch with other fundraisers and was able to share insights: “[it was] joyous because it was being around a lot of people, making new connections and thinking about things in different way. I really needed that” (FR9).

## 5 DISCUSSION AND CONCLUSION: RESILIENCE CAPABILITIES IN ARTS AND CULTURE FUNDRAISING

In this article, we examined the challenges brought about by the Covid‐19 pandemic to arts and culture nonprofits in the UK, and how fundraisers acted on those. In our analysis we found evidence that fundraisers were adept at building cognitive, behavioral, and relational capabilities, which supports our initial theoretical framework. The novelty of our framework lies in the way we applied literature on resilience capabilities in nonprofit and forprofit environments to the work of nonprofit fundraisers whose ability to raise income from face‐to‐face fundraising was severely disrupted during the early period of the Covid‐19 pandemic. In what follows, we summarize our main findings, and contribution to the literature as well as examine the implications of our findings for fundraising policy and practice.

Our *first key finding* is that fundraisers demonstrated an exceptional ability to build *cognitive, behavioral, and relational capabilities* very quickly in response to the challenges brought about by the pandemic. This finding aligns with Lengnick‐Hall and Beck's (2005) and Lengnick‐Hall et al.'s (2011) description of cognitive capabilities as having a sense of purpose and vision to be applied to the resolution of problems. Examples of cognitive capabilities in fundraisers included a swift grasp and awareness of how the priorities and values of donors and funders had quickly changed, and of the implications of these for their fundraising practice. They also displayed an acute awareness of the need to utilize digital technologies, such as social media, or other online platforms, to engage with audiences and supporters at a time when face‐ to‐face fundraising and cultural content offer were not an option. Such cognitive capabilities were converted into newly adapted ways of working in the form of *behavioral capabilities*, which we initially described as practical alternatives to steer a “dramatically different course of action” (Lengnick‐Hall & Beck, 2005, pp. 750–751). We found examples of fundraisers making new strategic decisions, such as to continue working with existing donors, and to create alternative avenues of income generation by delivering an online cultural offer. Existing skills such as perseverance and adaptability were mentioned as being particularly useful; helping fundraisers develop new ways of working that were more reactive and less planned, involving fewer but perhaps more pressing targets and timelines. The ability to deal with rejection was referred to as a positive skill and even described as a form of resilience, which had proved very useful during the pandemic. Finally, *relational capabilities* also pervaded in our data analysis. Described as having access to and exchange of resources so that an organization can function most effectively during periods of adversity, participation in social networks, for example, can facilitate access to resources e.g. information, loans and gifts (Lengnick‐Hall & Beck, 2005). Our most clear example of relational capabilities was the way in which fundraisers sought the collaboration of existing funders to obtain financial support, or to change the conditions attached to existing funding. This was a crucial and strategic practice on the part of fundraisers at a time when individual donors' and businesses' ability to contribute to nonprofits was negatively affected. By working together with funders fundraisers managed to change the conditions attached to existing funding from restricted to unrestricted; this meant that if certain funds could only initially be used for certain projects, this condition was changed so that nonprofits were able to use the funds in ways that would help them deal with their current challenging environment (provided there was agreement between funders and nonprofits). Fundraisers were key in helping such financial resources become available.

Our *second key finding* expands on the classifications from the literature because it points at how fundraisers merged different capabilities into one. This is what we refer to as the deployment of cross‐capabilities. Our analysis has demonstrated that *emotion‐related capabilities* were accessed as a way of supporting the implementation of *behavioral capability building*. We found such evidence in fundraisers who worked with donors who had shown an emotional engagement with the organizations; donors who had demonstrated their loyalty to the organization in pre‐pandemic times, and who, thus, were very likely to be most responsive to supporting specific fundraising efforts during the pandemic. In so doing, fundraisers' deployment of behavioral capabilities, or deciding on the most efficient, alternative, course of action, was based on the understanding of existing emotional links between donors and the organization. This combination of emotion‐related/behavioral capabilities led fundraisers to strategizing in ways that were addressing the concerns of the organization as well as aligning with what was emotionally meaningful to donors. A similar example of emotion‐related capabilities deployed together with behavioral capabilities was found when fundraisers decided to adopt an honest approach to their relationships with external funders. This strategy was designed to involve funders in a process of honest and open relationship building with the expectation that they would support nonprofits' efforts to either re‐direct awarded income towards funding new pressing initiatives, or towards acquiring new income. Both examples, even though only representative of the small sample in our data, clearly show that further research is needed around emotions and organizations (Fineman, 2003), and more specifically of the specific emotional repertoires informing financial decision making and practice (Pixley, 2004). We argue that not all resilience capabilities are put at work equally; emotion‐related capabilities provided fundraisers with the building block, the cement to help them build a strong set of behavioral capabilities. This type of cross‐capability work demonstrates that resilience capabilities are not always isolated realms of practice carried out independently from each other, but that they also occur and come into existence in and through relation to each other.

### 5.1 Implications for practice and policy

Our research findings have implications for future developments in government policy around arts and culture nonprofit fundraising. A key finding was around the importance of using digital technologies as a fundraising tool. But our interview data highlighted the unequal ways in which fundraisers were able to pursue their new digital agendas, shedding light on the disenfranchisement experienced by some nonprofits. Key concerns were the lack of financial income to invest in new staff with digital skills; a lack of support from senior management to implement online fundraising appeals; and how to address the disadvantaged position of some beneficiaries and audiences who did not have digital access. Such findings resonate with the most recent digital skills report for the charity sector (Skills Platform and Amar, 2021), which highlights both a lack of digital skills, as well as the challenge of digital inclusion when it comes to engaging with beneficiaries who do not have the skills nor technology to access charities' services. However, our findings also shed light on the need to create policies that support the building of digital skills in nonprofit fundraising. Despite the series of government‐funded campaigns to build a “resilient” fundraising arts and culture sector in the UK, all nonprofit fundraisers interviewed concurred in saying that their organizations were ill‐prepared to cope with the challenges to suddenly adopt digital skills in their online fundraising and audience engagement strategies. This is reason for concern given the relatively recent government‐backed investment in arts nonprofit fundraising. Between 2012–19 the ACE distributed over £11.7.5 million in government funds with the sole aim of building fundraising capacity for new money, and to support skills in the sector.^4^ Overall, the idea was that such support would aid fundraising “become more sustainable, resilient and innovative.” However, even though altogether the campaigns helped leverage a further £11.1 million in philanthropic donations,^5^ new policy frameworks are needed that address how best to support the building of resilience capabilities. The research presented here indicates that fundraising during a pandemic poses new and unexpected challenges to fundraisers' work practices, and that despite their ability to “cope” in such dire circumstances, there are areas, such as digital fundraising/engagement, that with the right investment could lead to an increase in income generation. Such specifically designed policies are needed as they could provide long‐term responses to the fundraising challenges emerging from this pandemic and from future post‐pandemic crises and uncertainties.

## CONFLICT OF INTEREST

No conflicts of interest were incurred in the writing of this article.

## Data Availability

The data that support the findings of this study are available from the corresponding author upon reasonable request.

## Endnotes

^1^
At the time, the most significant expenditure to arts and culture came from local authorities, which together they invested £1.1 billion in theaters, museums and libraries – more than the Arts Council investment of around £700 million each year, and National Museums that receive £450 million.
https://www.theguardian.com/society/2019/jan/28/councils-say-more-arts-cuts-inevitable-amid-rising-social-car e‐need.

^2^

https://www.instituteforgovernment.org.uk/charts/uk-government-coronavirus-lockdowns.

^3^
These categories follow the NCVO's Almanac size categories, which were also used in the survey (authors 2021).

^4^
The program Catalyst: Evolve (2012–15) also funded a three‐year Arts Fundraising and Philanthropy program offering training, fellowships, coaching, and digital skills across the sector to encourage innovation in arts fundraising https://www.artscouncil.org.uk/funding/catalyst-evolve#section-5.

^5^
In 2017, the ACE invested over £400,000 in the Institute of Fundraising's Culture Sector Network in a program of learning, training, mentoring and peer‐to‐peer networking opportunities made available to arts and cultural fundraisers across England. https://fundraising.co.uk/2017/06/27/iof-cultural-sector-network-receives-431832-arts-council/.
